# Brusatol Enhances the Radiosensitivity of A549 Cells by Promoting ROS Production and Enhancing DNA Damage

**DOI:** 10.3390/ijms17070997

**Published:** 2016-06-24

**Authors:** Xiaohui Sun, Qin Wang, Yan Wang, Liqing Du, Chang Xu, Qiang Liu

**Affiliations:** Tianjin Key Laboratory of Radiation Medicine and Molecular Nuclear Medicine, Institute of Radiation Medicine, Chinese Academy of Medical Sciences and Peking Union Medical College, Tianjin 300192, China; sxhyx201408@163.com (X.S.); wangyan@irm-cams.ac.cn (Y.W.); dlq@irm-cams.ac.cn (L.D.); xuchang@irm-cams.ac.cn (C.X.)

**Keywords:** NSCLC, brusatol, Nrf2, ROS, DNA damage, radiosensitivity, ionizing radiation

## Abstract

NF-E2-related factor 2 (Nrf2) has been identified as a master regulatory factor in the protection of cells from oxidative and electrophilic stress. However, overexpression of Nrf2 in lung cancer may cause chemoresistance, as well as radioresistance. In this study, we examined the relationship between radioresistance and Nrf2 protein levels in H1299, A549, and H460 cells, and finally chose the A549 cell line to continue with due to its strong radioresistance and high Nrf2 protein levels. We found that the Nrf2 inhibitor, brusatol, could prevent the increase and accumulation of Nrf2 after exposure to irradiation. Additionally, following treatment with 80 nM brusatol, A549 cells became sensitive to irradiation, suffering severe DNA damage. Combination treatment with brusatol and ionizing radiation (IR) can distinctly increase the level of reactive oxygen species in A549 cells, causing a 1.8-fold increase compared with the control, and a 1.4-fold increase compared with IR alone. In fact, in the treatment with both brusatol and IR, lung cancer cell proliferation is halted, gradually leading to cell death. Because Nrf2 is closely linked to DNA damage repair, inhibiting the function of Nrf2, as in brusatol treatment, may increase the DNA damage caused by radiotherapy or chemotherapy, possibly enhancing the efficacy of chemotherapeutic drugs. Our study is the first to demonstrate brusatol’s ability to enhance the responsiveness of lung cancer cells to irradiation, and its potential application as a natural sensitizer in radiotherapy.

## 1. Introduction

Among all cancers, lung cancer has the highest mortality rate worldwide. Routine treatment often involves chemotherapy combined with radiotherapy. The latter can effectively inhibit cell proliferation and induce cell death in the cancer cells by overproducing reactive oxygen species (ROS) [1,2]. However, high expression levels of ROS scavenging enzymes, either intrinsic or acquired, can confer radiotherapy resistance [3,4,5]. This greatly affects the clinical outcome, and there is, therefore, an urgent need for combination therapies, which could overcome the major resistance.

NF-E2-related factor 2 (Nrf2) belongs to the cap ‘n’ collar (CNC) subfamily of basic leucine zipper transcription factors [6], and was originally identified as a master regulatory factor in the protection of cells from oxidative and electrophilic stress [7]. Under unstimulated conditions, Nrf2 is sequestered in the cytoplasm by the anchor protein Keap1, and maintained at a stable level by the ubiquitin-proteasome-dependent degradation system [8,9,10]. However, when exposed to oxidative, electrophilic, or xenobiotic stress, Nrf2 escapes from Keap1-dependent repression and translocates to the nucleus, where it directs a series of cell protective proteins, such as the antioxidant phase II detoxifying enzymes, by binding to antioxidant response elements (ARE) with small musculoaponeurotic fibrosarcoma protein (sMaf) [11,12]. Research has verified that somatic mutations of Keap1 or Nrf2 result in a gain of function of Nrf2 in both lung cancer patients and lung cancer cell lines [13,14,15]. Additionally, constitutive activation of Nrf2 contributes to resistance to chemotherapeutic drugs and radiotherapy [1,16]. Furthermore, recent studies have demonstrated that inhibition of the Nrf2-dependent antioxidant defense system confers sensitivity to chemotherapy and radiotherapy [17,18]. Therefore, novel drugs targeting Nrf2 might be used as an effective strategy to enhance the radiosensitivity of lung cancer cells, ultimately improving clinical outcomes for patients.

*Brucea javanica*, a plant distributed widely throughout Asia, especially in Southern China, has been used in traditional Chinese medicine for treating various diseases, including cancer, amoebic dysentery, and malaria [19,20,21]. Brusatol, a quassinoid that can be obtained from *Brucea javanica*, demonstrates a wide range of pharmacological activities, including antitumor, antimalarial, anti-inflammatory, antiviral, and insecticidal activities [22]. These functions are attributed to its two effects, inhibition of proliferation and induction of differentiation [23,24]. Recently, studies show that it can inhibit DNA and RNA synthesis and enhance the efficacy of chemotherapy by inhibiting the Nrf2 mediated defense mechanism [18]. Despite all this, the use of *Brucea javanica* is severely limited by its high cytotoxicity. However, some researchers have synthesized a series of brusatol analogues to investigate the relationship between its structure and activity, and to improve its medicinal profiles [22,25]. Based on this promising finding, along with the accumulating evidence of its anticancer properties, we expect brusatol may also enhance the efficacy of radiotherapy by inhibiting Nrf2.

## 2. Results

### 2.1. Comparing the Irradiation Sensitivity of Three Non-Small-Cell-Lung Carcinoma (NSCLC) Cell Lines

**Figure 1:**
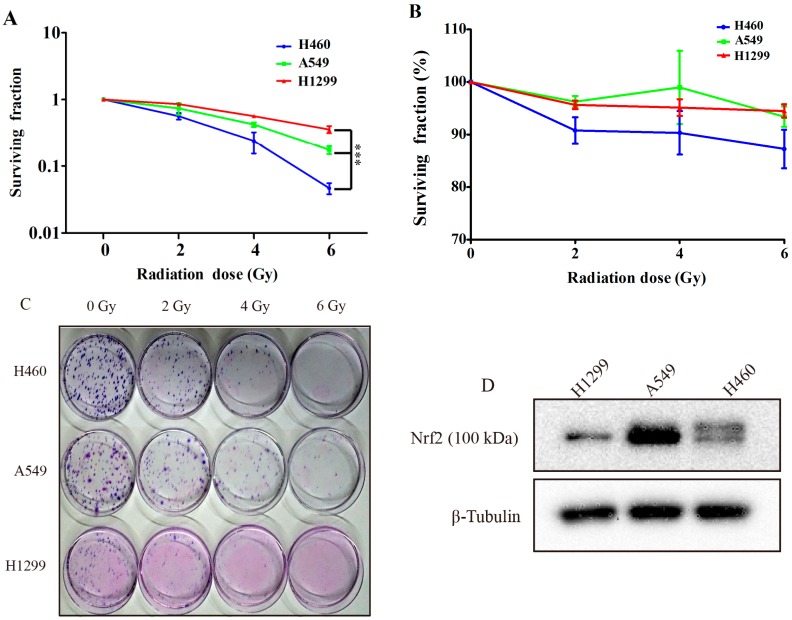
Differences in radiosensitivities and Nrf2 protein levels for three NSCLC cells. (**A**,**C**) H1299, A549, and H460 cells were exposed to the indicated dose of IR, 0, 2, 4, or 6 Gy, and cultured for 1–2 weeks with fresh media provided every 3 days. Colonies with more than 50 cells were counted. *** *p* < 0.001 , H1299 and A549 compared with H460; (**B**) The fraction of surviving cells as detected by MTT assay; and (**D**) protein levels in H1299, A549, and H460 cells.

To clarify the different irradiation sensitivities of the three NSCLC cells and to examine the relationship between irradiation sensitivity and their Nrf2 protein levels, we performed colony formation assays, 3-(4,5-Dimethylthiazol-2-Yl)-2,5-Diphenyltetrazolium Bromide (MTT) assays, and western blotting. We calculated the colony formation rate of the cells after about two weeks of exposure to 0, 2, 4, and 6 Gy γ-irradiation (Figure 1A,C). The H460 cell line was obviously more sensitive to γ-irradiation than the H1299 and A549 cell lines. However, the colonies of the H1299 and A549 cells were always smaller, looser, and lighter than the H460 colonies, which was especially true in the H1299 cells. Although there was a good amount of variation in the sample error in the MTT assay data, the trend in irradiation sensitivity was parallel with that observed in colony formation assay (Figure 1B).

Studies have demonstrated that an Nrf2 gain of function is present in NSCLC cells and is responsible for conferring radioresistance [16]. Thus, we set out to detect the total protein of three NSCLC cells using western blotting in order to determine whether or not the different Nrf2 protein levels correlated with irradiation sensitivity. The results (Figure 1D) showed that the A549 cells had the highest levels of Nrf2 protein, whereas the total Nrf2 protein quantity in H460 cells is much less. These findings are in parallel with the radiosensitivity data. Surprisingly, H1299 cells do not possess the highest level of Nrf2 protein, contrary to the observed relationship for their radioresistance. We propose that this might be due to the P53 deficiency of H1299 cells. Based on the results of this study, we chose H1299 and A549 cells, both of which have serious radioresistance, as the subjects for our further research.

### 2.2. Brusatol Inhibits Activation of Nrf2 in A549 Cells after Exposure to Irradiation

**Figure 2:**
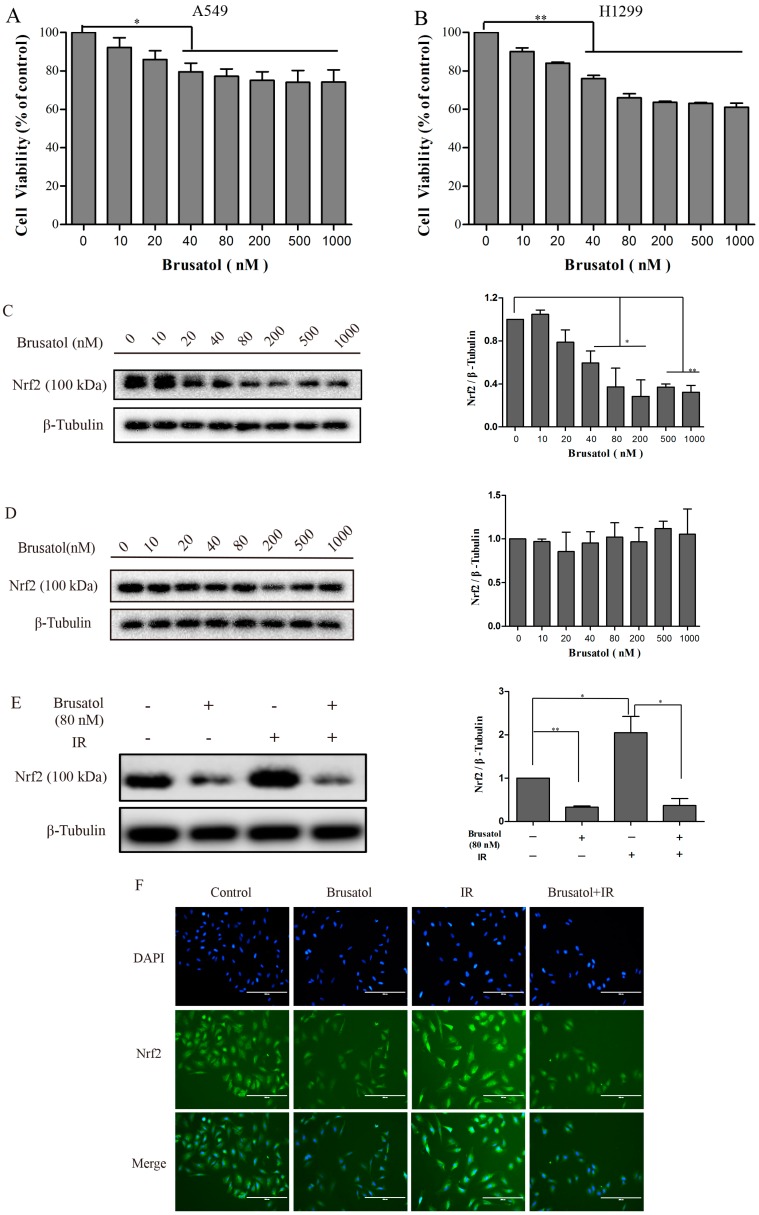

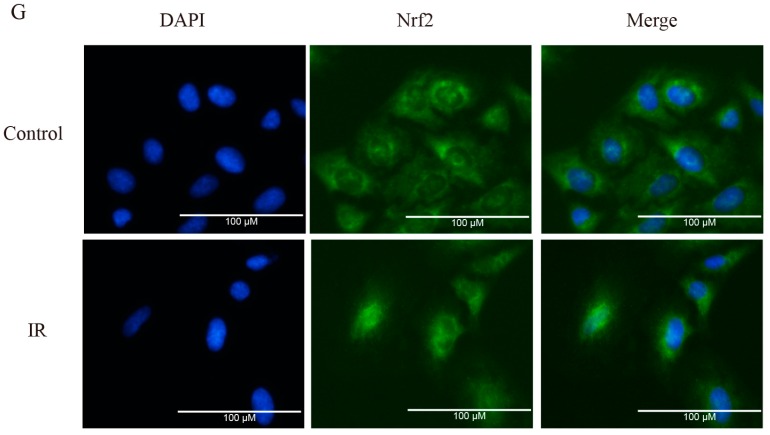
The effects of brusatol on A549 and H1299. (**A**,**B**) The cytotoxicity of brusatol was determined under the same conditions for the MTT method; (**C**,**D**) A549 and H1299 cells were treated with the indicated concentrations of brusatol, and were collected after 6 h. Then we extracted total proteins for Western blot analysis; (**E**) A549 cells were treated with, or without, 80 nM brusatol for 4 h, then exposed to 6 Gy γ-irradiation or not. Two hours later, we extracted total proteins for Western blot analysis; (**F**,**G**) The Nrf2 protein level of A549 cells was determined under the same experimental conditions as in (**E**) using the Immunofluorescence method. (The scale bars in Figure 2F are 200 µM) ** *p* < 0.01, * *p* < 0.05.

Brusatol has recently been identified as an Nrf2 signaling inhibitor, reducing tumor burden and enhancing the efficacy of chemotherapeutic drugs, both in vivo and in vitro [18]. To study the effect of brusatol on improving the sensitivity of the cells to irradiation, we tested the cytotoxicity and suppression efficiency of Nrf2 in H1299 and A549 cells (Figure 2). Cells were treated with brusatol for 24 h and cell viability was measured (Figure 2A,B). We found that brusatol had high toxicity in both A549 and HI299 cells.

Western blotting revealed that brusatol exerted a dose dependent inhibition of Nrf2 protein levels in the A549 cells (Figure 2C). A significant decrease in Nrf2 protein level was observed after treatment with 20 nM of brusatol, with the most effective inhibition function observed at 80–200 nM. However, brusatol had no obvious effect on the Nrf2 protein level of H1299 cells (Figure 2D). As a next step, we focused on A549 cells, and explored whether or not brusatol contributes to increasing their radiosensitivity. Irradiation induces an increase in the protein level of Nrf2 in A549 cells. This IR-induced increase in Nrf2 protein expression may be in response to an excessive production of ROS, but this effect can be significantly downregulated by combining IR with brusatol treatment (Figure 2E). Immunofluorescence assays showed that 6 Gy γ-irradiation could result in an obvious induction of Nrf2 nuclear accumulation and an increase in the fluorescence intensity of Nrf2 protein, as compared with the control group. In contrast, the fluorescence intensities of Nrf2 in the groups treated with brusatol were faint, and the Nrf2 protein was uniformly distributed in the cytoplasm (Figure 2F,G).

### 2.3. Brusatol Treatment Increases the Radiosensitivity of A549 Cells by Increasing the Level of ROS, Leading to Serious DNA Damage

**Figure 3:**
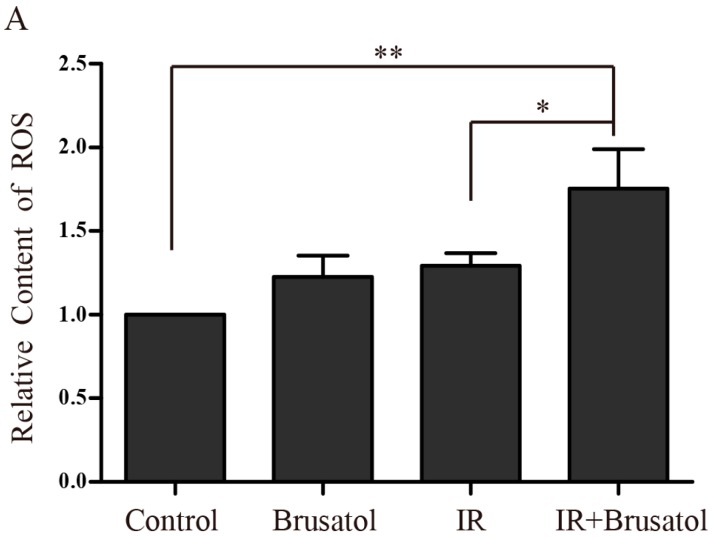

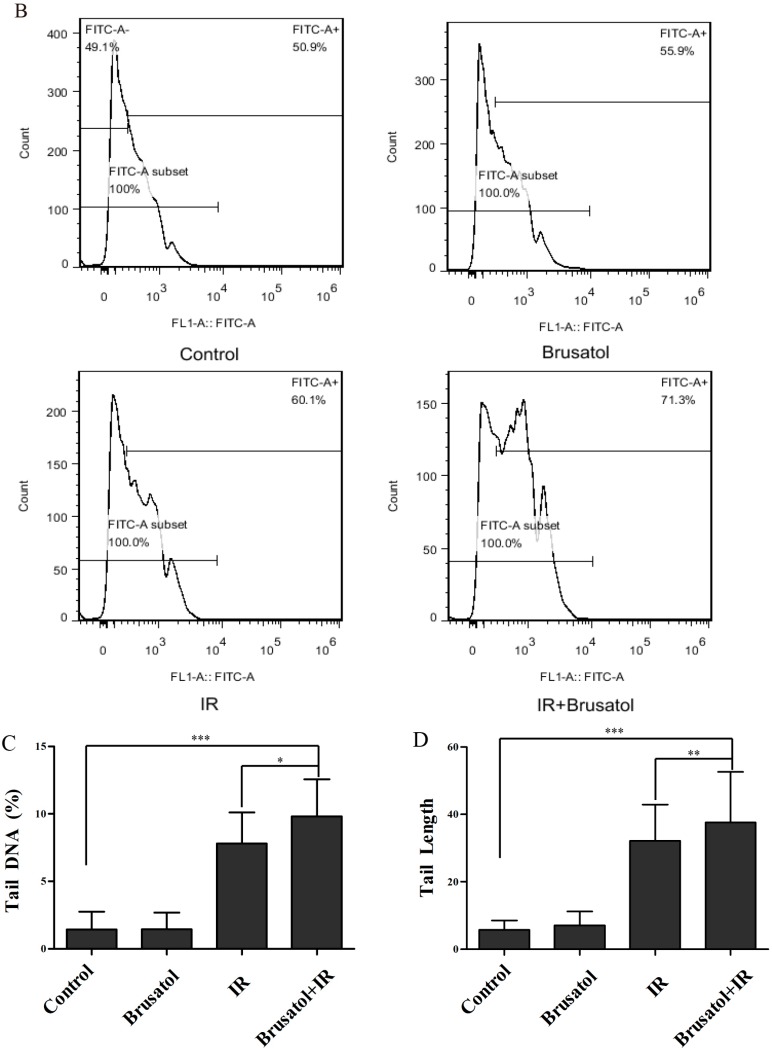

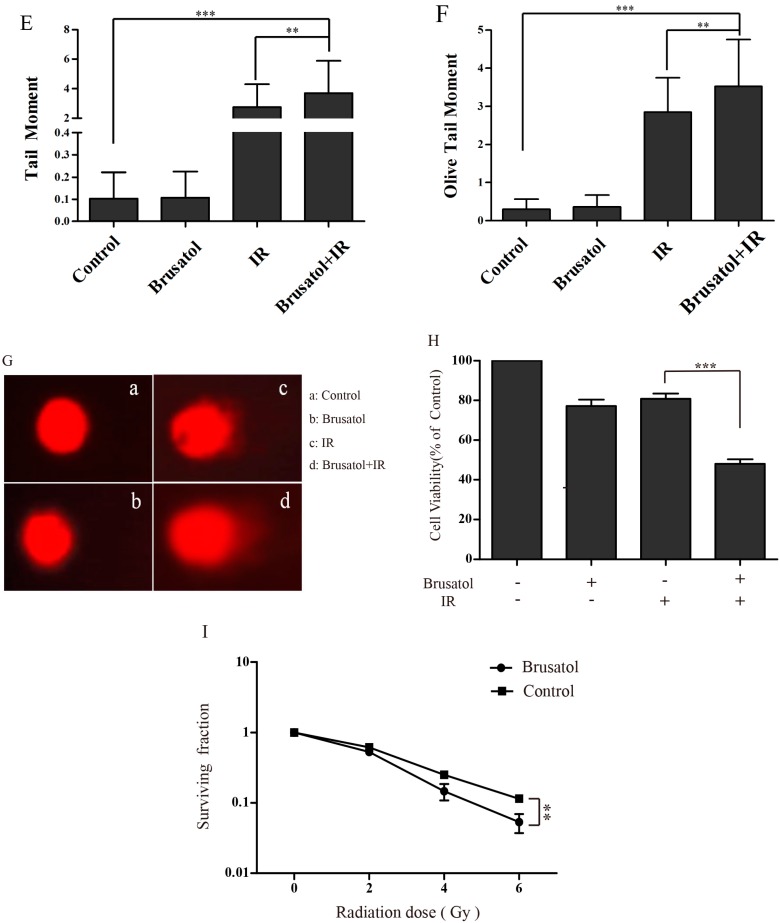
Brusatol enhances the radiosensitivity of A549 cells. (**A**,**B**) Detection of ROS levels by the fluorescence indicator H2DCFH-DA 6 h (**A**) or 24 h (**B**) after exposure to 6 Gy γ-irradiation, using flow cytometry (FCM); (**C**–**G**) Detection of DNA damage by comet assay; (**H**) A549 cells were treated with or without 80 nM brusatol for 4 h, then were exposed to 10 Gy γ-irradiation or not. 24 h later, cell viability was measured by MTT assay; (**I**) A549 cells were treated with, or without, 80 nM brusatol for 4 h, then were exposed to the indicated dose of γ-irradiation. Colonies containing more than 50 cells were counted 1–2 weeks later. *** *p* < 0.001, ** *p* < 0.01, * *p* < 0.05.

To determine if the decrease in Nrf2 protein induced by brusatol contributes to increased ROS accumulation in A549 cells, we measured intracellular ROS levels after IR, using the fluorescence indicator H_2_DCFH-DA. Combination treatment of A549 cells with 80 nM brusatol and IR can distinctly increase the level of ROS, 4 h after 6 Gy irradiation. Fluorimetric quantification of the change in ROS showed that combination treatment caused an approximate 1.8-fold increase in the level of ROS compared with control, and a 1.4-fold increase compared with IR alone. Additionally, brusatol alone had an effect on the generation of ROS (Figure 3A). Twenty-four hours after 6 Gy irradiation, the combination treatment group had more cells containing ROS, compared to the median level in the IRalone control group (Figure 3B).

We used a single cell gel assay to detect the DNA damage caused by IR. Analysis of the percent of tail DNA, tail length, tail moment, and olive tail moment, yielded the result that brusatol alone had no significant effect on DNA, but that IR caused obvious damage to DNA. As predicted, combination treatment further aggravated the damage (Figure 3C–F). The visual comet image is shown in Figure 3G. Brusatol combined with IR caused higher percent of tail DNA and longer tail length than IR alone, whereas brusatol had little effect on A549 cells.

Data from clonogenic survival assays and MTT assays revealed that brusatol enhanced the radiosensitivity of A549 cells (Figure 3H,I). During cell culture, we found that IR alone could significantly inhibit cell proliferation, but did not have the same effect on cell survival, which increased even greater than that of the control group. However, when treated with both brusatol and IR, cell proliferation is halted, to a degree much less than that of the control cells, eventually leading to gradual death. This phenomenon could also be found in the Immunofluorescence (Figure 2G).

## 3. Discussion

A major challenge in the attempt to cure cancer successfully is the radioresistance that is acquired during radiotherapy. Fortunately, it has been demonstrated that Nrf2, a major regulator of redox homeostasis, plays an important role in radioresistance and the gain function of Nrf2 in NSCLC cells confers radioresistance. Downregulation of Nrf2 by shRNA increased radiosensitivity of A549 cells [16]. Emerging investigations also supported the hypothesis that activation of Nrf2 signaling promoted a prosurvival response in irradiated cells, such as fibroblasts, bronchial and breast epithelial cells, and glioblastoma cells [26]. Therefore, our first step was to compare the radioresistance of three representative lung cancer cell lines, H1299, A549, and H460. We also examined the Nrf2 protein expression levels in the cell lines, to clarify the relationship between Nrf2 and radioresistance among the different cells. In fact, it is the high level of Nrf2 in A549 cells that makes them more resistant than H460 cells. However, we only compared three cell lines, and the H1299 cells presented a crosscurrent result, which could be attributed to P53 deficiency. In order to clearly and definitively identify the relationship between Nrf2 and radioresistance in NSCLC, more cell lines should be examined.

Nrf2 is a nuclear transcription factor that protects cells by integrating cellular stress signals, directing various transcriptional programs [27], as well as involvement in various cellular processes, such as proliferation, differentiation, migration, apoptosis, and angiogenesis [28,29]. Pharmacological development of drugs targeting Nrf2 is expected to prevent diseases caused by oxidative or inflammatory stress and cancers. A wealth of evidence supports Nrf2’s role as an effective target in the attempt to cure cancer. Elevated Nrf2 protein levels are observed in cancers, such as lung, head and neck, gall bladder, and pancreatic cancer [30,31], and a gain of Nrf2 function enhances cell proliferation and confers radioresistance and chemoresistance in these cancer types [1,16,32,33]. To improve the efficacy of cancer treatments, researchers have developed a series of strategies regulating the level of Nrf2 protein. For example, alkaloid trigonelline can inhibit Nrf2 function and promote apoptosis in pancreatic cancer cells through regulating proteasome gene expression and activity [34]. Additionally, IM3829, an effective blocker of Nrf2, increases the radiosensitivity of NSCLC [17]. Brusatol has been researched as an Nrf2 inhibitor that can enhance the efficacy of chemotherapy in A549 cells [18]. However, no studies have focused on brusatol’s function on other NSCLC cell lines, and whether or not brusatol combined with radiotherapy is more effective than radiotherapy alone is not yet clear. In our study, we found that brusatol can enhance the effectiveness of radiotherapy, inhibit the function of Nrf2 and increase generation of ROS in A549 cells. Vartanian S et al. reported that the activity of brusatol was not restricted to Nrf2, but rather, functioned as a global protein synthesis inhibitor [35]. So, the radio-sensitization effect of brusatol might be associated with the inhibition of Nrf2, which might not be the only mechanism for the radio-sensitization.

To our surprise, brusatol had no significant inhibitory effect on H1299 cells, in which P53 is deficient. Dongmei Ren et al. demonstrated that brusatol inhibits Nrf2 protein levels by enhancing ubiquitination and degradation, which is not directly related to the Keap1 protein level, but to decreased ubiquitination of Keap1 [18]. Adedamola Olayanju et al. also found that brusatol provokes inhibition of Nrf2 signaling and function, and that the effect is specific to Nrf2, independent of hypoxia inducible factor α, cyclinA, and p53 protein levels [36]. On the other hand, it has been demonstrated that there is cross-talk between P53 and Nrf2 [37]. Despite these findings, the mechanism by which brusatol inhibits Nrf2 has yet to be identified clearly. When considering our findings in concert with published researches, we suspect that P53 may play an important role in Nrf2 ubiquitination and degradation, and we aim to explore this topic in our future studies.

Nrf2 can also promote survival of cells that have been exposed to irradiation [26]. This protective effect may be owed to Nrf2’s regulation of DNA repair gene expression. Some researchers found that Nrf2 stimulates breast cancer susceptibility gene 1 (BRCA1) expression by binding to ARE [38]. Furthermore, others reported that an increase in Nrf2 function promotes the expression of p53-binding protein 1, which induces DNA repair signaling and protects cells from irradiation [39]. In our studies, the protein level of Nrf2 increased following irradiation, and Nrf2 was found mainly gathered in the nuclei, much like DNA repair proteins, such as the epidermal growth factor receptor [40]. We also found that when we treated with brusatol prior to irradiation, the cells suffered more DNA damage than in the control group. Thus, we concluded that Nrf2 is closely linked to DNA damage repair and inhibiting the function of Nrf2 could serve as an effective strategy for increasing the DNA damage caused by radiotherapy or chemotherapy.

The goal of this study was to identify the Nrf2 inhibitor, brusatol, as a novel radiosensitizer, which could overcome the radioresistance of lung cancer cells by promoting ROS production and increasing DNA damage. In line with our prospection, dual treatment with brusatol and radiotherapy could effectively inhibit the proliferation of lung cancer cells. However, the application of brusatol as a radiosensitizer should be approached cautiously, as its high toxicity may hinder its effective use. It is, therefore, necessary to further investigate brusatol derivatives, which may circumvent this toxicity, and be more suitable for treatment as radiosensitizers.

## 4. Materials and Methods

### 4.1. Reagents and Cells Culture

The human NSCLC cell lines A549, H460, and H1299 were purchased from the Institute of Basic Medical Sciences Chinese Academy of Medical Sciences & School of Basic Medicine Peking Union Medical College.

Roswell Park Memorial Institute (RPMI) 1640 culture media was obtained from HyClone (Logan, UT, USA). Fetal bovine serum (FBS) was obtained from Gibco (Grand Island, NY, USA). Bicinchoninic acid (BCA), MTT, 2,7-dichlorodihydrofluorescein diacetate(H_2_DCF-DA), Giemsa stain, and 4′,6-diamino-2-fenilindol (DAPI) were obtained from Sigma (St. Louis, MO, USA). Brusatol was obtained from Tauto Biotech (Shanghai, China). The anti-Nrf2 antibody and anti-β-Tubulin mouse monoclonal antibody were obtained from abcam. Fluorescein conjugated affinipure goat anti-rabbit IgG (H+L) was obtained from ZSGB-BIO (Beijing, China).

The cell lines A549, H460, and H1299 were maintained in RPMI 1640 (Hyclone) supplemented with 10% FBS and a 100 U/mL penicillin, 100 μg/mL streptomycin solution, and grown at 37 °C in a humidified 5% CO_2_ atmosphere.

### 4.2. Protein Extraction and Western Blotting

Cancer cells were lysed in RIPA buffer at 4 °C for 20–30 min and centrifuged at 12,000× *g* at 4 °C for 15 min to obtain total protein lysates for immunoblot analysis using 40 μg of total protein lysate resolved on 10% SDS-PAGE gels. Proteins were then transferred onto PVDF membranes, and subjected to immunoblotting. All primary and secondary antibodies were diluted in TBST containing 5% nonfat dry milk. Primary antibody incubation was performed at 4 °C overnight, and secondary antibody incubation was performed at room temperature for about 2 h. Immunoreactive proteins were visualized with enhanced chemiluminescence reagents, according to the manufacturer’s recommendations.

### 4.3. Cell Irradiation

An irradiator equipped with a Cs-137 (Gammacell-40) source purchased from Atomic Energy Co. (Atomic Energy of Canadian Inc., Mississauga, ON, Canada). Sealed, sterile cell culture plates or dishes were placed in the center of the irradiation chamber and exposed to the radioactive, source, delivering uniform irradiation at a dose rate of 1.02 Gy·min^−1^.

### 4.4. MTT Assay

Cells were plated in 96-well plates at a density of 5 × 10^3^ cells/well in triplicate, and then treated with the indicated concentrations of drugs and doses of irradiation, followed by a 24 h incubation. MTT solution (10 μL) was added to each well and incubated for 4 h before complete removal of medium from each well, and addition of dimethyl sulfoxide (DMSO) (150 μL). The absorbance at 570 nm was measured using a microplate reader.

### 4.5. Detection of Intracellular ROS

Intracellular ROS levels were measured using H2DCFH-DA (Molecular Probes, Invitrogen, Waltham, MA, USA). Briefly, cells were treated with or without brusatol 3 h prior to irradiation, then treated with 10 μM H2DCFHDA for 20–30 min, and finally washed with phosphate buffer saline (PBS) before trypsinization. After detachment, the cells were collected, washed twice, and resuspended in 500 μL PBS. Fluorescence was detected using a flow cytometer at excitation/emission wavelengths of 488/525 nm.

### 4.6. Colony Formation Assay

H1299, H460, or A549 cells were plated at the same densities (400 cells per well) in 60-mm dishes in triplicate, treated with brusatol for 2 h, and exposed to the indicated doses of ^137^Cs γ-radiation (1.02 Gy/min). Two hours after irradiation, the medium was replaced with fresh medium without any drug treatment. The cells were then incubated for about 2 weeks, with a change of medium every 2 days. Finally, the cells were stained with Giemsa, and colonies containing more than 50 cells were counted.

### 4.7. Immunocytofluorescence

A549 cells (2.0 × 10^3^ cells per well) were cultured in 96-well plates with sterilized coverslips and treated with or without the brusatol/IR treatment. After 24 h irradiation, these cells were washed 3times with PBS, and fixed with 4% paraformaldehyde for 20 min at room temperature. They were then washed 3 times with PBS for 5 min, and permeabilized in 0.3% Triton-100/PBS for 15 min. Following this preparation, the cells were then subjected to immunodetection. All washes were performed 3 times with PBS for 5 min. First, the cells were incubated with 10% goat serum/PBS for 30 min at room temperature and then incubated with the polyclonal anti-Nrf2 antibody at 1:1000 in 10% goat serum/PBS overnight at 4 °C. Cells were then washed and incubated with the secondary anti-rabbit IgG antibody conjugated with FITC-isomer1 fluorochrome diluted 1:100 in 10% goat serum/PBS for 1 h. Finally, the cells were washed and incubated with 50 μL of 0.5 μg/mL DAPI for 5 min at room temperature to stain the nuclei. Fluorescence images were visualized with a fluorescence microscope.

### 4.8. Single Cell Gel Assay (Comet Assay)

After exposure to 4 Gy irradiation, cells were digested and collected immediately, washed twice with PBS, and suspended in PBS at a density of 4–5 × 10^5^ cells/mL. The comet slides were coated with 500 μL of 0.75% normal-melting-point agarose/PBS. Once the first layer of normal-melting-point agarose was coagulated, a mixture of 70 μL of 0.75% low-melting-point agarose/PBS and 30 μL of cell suspension was applied as the second layer. The comet slides were then immersed in cold fresh lysis solution (2.5 M NaCl, 10 mM Tris base, 1% *N*-sodium lauryl sarcosinate, 30 mM Na_2_EDTA, 10% DMSO, 1% Triton X-100) for 2.5 h at 4 °C. Next, the comet slides were immersed in TBE buffer for 20 min in a horizontal electrophoresis tank and electrophoresis was performed at 30 V for 20 min in TBE buffer, followed by neutralization for 20 min. The slides were then rinsed twice with PBS and stained with ethidium bromide. Finally, the comet slides were viewed with a fluorescence microscope and data were collected with a digital imaging system and analyzed with CASP software (Wroclaw, Poland).

### 4.9. Statistical Analysis

Each experiment was performed at least 3 times, and results are shown as means ± standard deviation (SD). The student’s *t*-test was used to analyze the statistical significance of the results. Between groups, differences with *p*-values <0.05 were considered statistically significant.
